# Effects of portion size on chronic energy intake

**DOI:** 10.1186/1479-5868-4-27

**Published:** 2007-06-27

**Authors:** Robert W Jeffery, Sarah Rydell, Caroline L Dunn, Lisa J Harnack, Allen S Levine, Paul R Pentel, Judith E Baxter, Ericka M Walsh

**Affiliations:** 1 Division of Epidemiology and Community Health, School of Public Health, University of Minnesota, MN 55454-1015, USA; 2 Department of Food Science and Nutrition, College of Human Ecology, College of Agriculture, Food and Environmental Sciences, University of Minnesota, MN 55454-1015, USA; 3 Hennepin County Medical Center and Department of Medicine, University of Minnesota, USA

## Abstract

**Background:**

This study experimentally examined the effects of repeated exposure to different meal portion sizes on energy intake.

**Methods:**

Nineteen employees of a county medical center were given free box lunches for two months, one month each of 1528 and 767 average kcal. Foods were identical in the two conditions, but differed in portion size. Meals averaged 44% calories from fat. Participants self-reported how much of each lunch was eaten. Unannounced 24-hour dietary recalls were also conducted by phone twice per week during each exposure period.

**Results:**

Mean energy intake at the lunch meal was 332 kcal/day higher in large lunch than in small lunch periods (p < .001). Mean 24-hour energy intake was 278 kcal/day higher in large versus small lunch periods (p < .001). There was no evidence of compensation over time. Average weight change over the month of large and small lunches was 0.64 ± 1.16 kg and 0.06 ± 1.03 kg, respectively, about what would be expected with the observed differences in energy intake.

**Conclusion:**

This study suggests that chronic exposure to large portion size meals can result in sustained increases in energy intake and may contribute to body weight increases over time.

## Background

Over the last 20 to 30 years, there have been dramatic increases in the prevalence of obesity in all segments of the US population [1]. Exact causes of this trend remain unclear, but increased food intake and/or decreased physical activity are certainly the proximal causes. Most scientists agree that factors driving these behavior changes are more likely to be environmental, broadly defined than biological. Data on aspects of the environment that have co-varied in rough temporal symmetry with rising obesity rates have suggested a number of possible contributing factors, including changes in the cost of food, increased food marketing, and increased availability of sedentary entertainment, e.g., television. One change in the food marketplace that has attracted particular attention is food portion sizes, which have grown in an increasing number of food products sold in stores, vending machines, and restaurants, as well as in foods served at home [2].

It has been noted that physiologic regulatory mechanisms are much more efficient at signalling under-consumption than over-consumption of energy [3]. Thus, it makes sense that chronic exposure to portion sizes that exceed energy needs might promote chronic over-consumption and excess weight gain. Studies directly investigating the relationship between portion size and energy consumption in single-meal settings have found that large portion sizes significantly increase energy intake among adults [4]. In one study in a restaurant setting, adults purchasing a larger entrée portion increased entrée energy intake by 43% and of the entire meal intake by 25% [5]. In a study of undergraduate students, the larger the portion size of a served meal, the more calories were consumed [6]. In young children, doubling the age-appropriate portion at lunch of an entrée increased total energy intake by 15% [7]. Energy intake in snacks also increases as package size increases [8].

Despite demonstrated differences in energy consumption, research has shown no substantial variations in reports of hunger or satiety between subjects served standard or large portion sizes [9]. Subjects' perception of their energy intake when eating standard versus large portion sizes indicates that they are largely unaware that larger portion sizes induce higher energy intake [10].

Whether chronic exposure to larger portion sizes would result in sustained increases in energy intake or contribute to long-term weight gain, however, remains unclear. Rolls and colleagues [8,11,12] have conducted a series of studies showing that the effects of larger and smaller portion sizes on energy intake are sustained for 48 hours. Whether compensation for exposure to different portion sizes would occur spontaneously over longer time periods or whether changed portion sizes would lead to weight gain or loss has not been demonstrated.

To assess whether chronic exposure to larger portion sizes can cause chronic increases in energy consumption, the present study presented study subjects with meals of different portion sizes in a naturalistic setting for a sustained period of time and observed energy intake at meals, daily energy intake overall, and body weight. It was hypothesized that chronic exposure to large portion sizes compared to small portion sizes would result in higher energy intake at meals, higher average total energy intake per day, and possibly an increase in body weight.

## Methods

### Participants

Participants for the present study were recruited from employees of a community medical center by posting fliers on bulletin boards, in-person recruitment outside the center cafeteria, e-mail newsletter announcements, and table tents. The sample was restricted to women in an effort to reduce between subject variability and to simplify the logistics of food provision. Eligibility criteria were age 18 to 40, employment at the medical center, self-reported BMI between 18.5–40.0, not pregnant or recently having given birth, not actively dieting to control weight, not more than three days a week of regular moderate or vigorous physical activity and willingness to consent to the conditions of study participation. Study participants were offered $200 dollars for completing all study measures, paid in three installments. Women volunteers had a mean age of 33 (± 5.2) years, 45% were married, 65% reported having at least a 4-year college degree, and 80% were white. Mean BMI at baseline was 28.9 (± 7.8).

### Design

The study employed a within-person, randomized crossover design comparing the effects of providing free box lunches of different portion sizes 5 days per week for four consecutive weeks on energy intake and body weight. The study protocol included a baseline assessment, a 2-week run-in period, a 4-week period in which the participants received a free box lunch daily with small or large portion sizes, a 2-week washout period during which weight was assessed again, a second 4-week period with free lunches of the opposite portion size as in the first month, and finally a follow-up assessment. Twenty women in all were recruited for the study. Half were randomized to each order of lunch presentation. The sample size was chosen to detect differences in energy intake and was similar to sample sizes used in similar, though more tightly controlled laboratory feeding studies [8,10,12]. We did not expect to see statistically significant differences in body weight with this sample size.

### Procedures

This research was approved by the University of Minnesota and the Minneapolis Medical Research Foundation institutional review boards. Prospective study participants were screened for eligibility by phone and they then attended orientation sessions at which procedures were described, written consent was obtained, and baseline measures were collected. Candidates were told that the study was being conducted to assess factors influencing eating habits and the feasibility of providing daily box lunches. No specific mention was made of portion size or energy intake as study objectives until the final follow-up visit at which time the study purpose was disclosed. Because all participants received both sets of lunches, and because individuals receiving different portion size lunches were not prevented from interacting during the study, many became aware of the portion size manipulation as the study progressed, but most remained unaware of the study's intent. Although blinding to the portion size manipulation was considered, it was not attempted, in part because we thought it could be difficult to do while keeping the study exposures naturalistic, and in part because we thought that any bias related to knowledge of portion size would probably work against rather than for observing a portion size effect on intake.

The lunches used in the study were prepared by a local caterer with guidance from study nutrition personnel. A rotation of seven different lunches was used. The contents were typical lunch items that included a main course, side dish, dessert, and a drink. Main courses were sandwiches or salads. Side dishes were fruit or vegetable salad, chips, or bread depending on the main course. Desserts were cookies or bars. Drinks were water, Coke, or Sprite. Lunches of different energy content had the same selection of items and as close as possible to the same relative distribution of calories in the different items. We targeted 750 kcal for the small lunch in the belief from other data sets that it would approximate a normal lunch, about 1/3 of average energy intake per day in the US [13]. Fifteen hundred (1500) kcal was the target for the large lunch, chosen in the belief that it would be much larger than a normal lunch. All meals were pretested prior to study implementation by weighing and measuring all items and entering values obtained into the Nutrition Data System for Research (NDS-R) software, version 5.0_35 (2004), developed by the Nutrition Coordinating Center (NCC), University of Minnesota, Minneapolis, MN [14]. The obtained average value for small lunches was 767 kcal. Large lunches averaged 1528 kcal. Meals average 44% calories from fat. Lunches were delivered to the medical center study office daily during the study. Study participants picked them up at that location. For quality control purposes, one "extra" meal of each type was delivered each day for the first week of the study and each food item was inspected and weighed by research assistants to assure that portion size differences were being adhered to. In subsequent weeks, sample meals were visually inspected each day.

### Measures

Height and Weight: Body weight was measured on a calibrated electronic scale to the nearest 0.1 kg at three points in time: baseline, immediately after the first month of free lunches, and at the follow-up evaluation immediately after the second month of free lunches. Height was measured at baseline using a wall-mounted ruler.

Two kinds of dietary information were collected during the study. First, dietary intake at lunch was assessed by having study participants complete a self-administered questionnaire after each lunch in which they estimated the proportion of each food item eaten using a visual analogue scale. Participants received these questionnaires along with their lunch boxes and returned them either by interoffice mail or directly to the study office. They also reported any food items eaten at lunch that were not from their lunch box. These data were entered into the NDS nutrient analyses program described above [13]. Estimates of energy and macronutrient intake were calculated. The second diet assessment method was to conduct two 24-hour dietary recalls by telephone on randomly selected days for each participant during each of the lunch intervention weeks. These data were also entered into the NDS dietary assessment system in the same way as the lunch data and the same nutrients calculated.

Physical activity was assessed at baseline using an instrument developed by Jacobs [15], and twice in each experimental period and once during the washout period by asking participants to report type and duration of any leisure-time physical activity done in the last 24 hours. Daily energy expenditure was estimated in minutes per day by looking up reported activities in a compendium of physical activity compiled by Ainsworth [16].

### Analyses

Statistical analyses of the data from this study were done using SAS version 8.7. The analyses of the meal size manipulation on kilocalories consumed and on percent calories from fat at the lunch meal and per day were carried out using a general linear mixed model analysis, controlling for order of lunch presentation and physical activity as fixed effects and participant as a random effect. The weight change outcome was also analyzed using a general linear mixed model without controlling for physical activity.

## Results

Completion rates in the study were good. One participant had to withdraw from the study very early due to a health problem. In the remaining 19 participants, completion of weight assessments was 100%, of post-lunch meal reports was 91%, and of telephone recalls of diet and activity was 98%.

**Figure 1:**
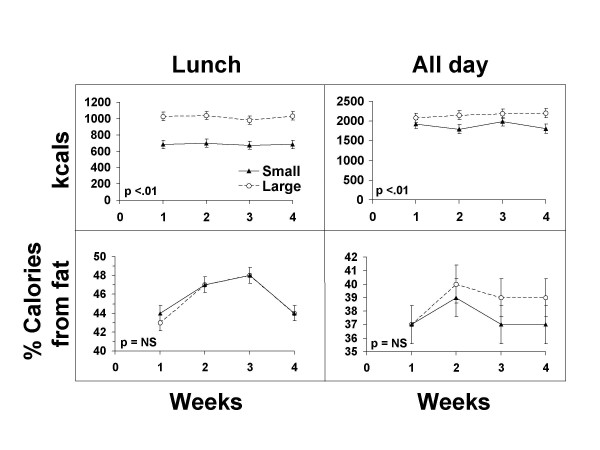
Box Lunch Study: Effect of treatments on energy and percent fat intake at lunch and per day.

Results of the study with regard to the effects of portion size on energy and fat intake are shown in Figure 1. Average reported energy intake at the lunch meal was 687 kcal during the small lunch period and 1019 kcal during the large lunch period (p < 0.0001). Total daily energy intake averaged 1875 kcal on small lunch days and 2153 kcal on large lunch days (p < 0.0001). There was no indication of compensation for increased lunch intake over the four weeks of exposure to large portion sizes. The lunches served were high in fat (about 45% of calories). The percent of calories from fat eaten at lunch did not differ by portion size condition. Additional analyses also indicated that order of portion size presentation did not influence food intake.

In theory, a 278-kcal/day elevation in energy intake would result in a 0.72-kg increase in body weight over 20 days if there were no compensation on days not observed (i.e. weekends). We observed average weight gains of 0.06 ± 1.03 kg during the month of small lunches and 0.64 ± 1.16 kg during the month of large lunched, figures remarkably close to theoretical prediction. Due to our small sample size, the within-person difference in weight change during large and small lunch months did not reach conventional levels of statistical significance (p = 0.13).

## Discussion

This study showed that chronic exposure to larger portion sizes in free-living populations can induce sustained increases in energy intake and suggests that the effects of portion size may be powerful enough to affect rate of weight gain over time. The study was of too limited a sample size and duration to provide definitive proof that chronic exposure to larger portion sizes increases risk for undesirable weight gain or that people exposed to them would not "spontaneously" bring their weight under better control over time. However, it is believed that this demonstration is a meaningful addition to the accumulating body of evidence supporting the idea that offering larger portion sizes as part of food marketing strategies could have deleterious effects on population obesity rates if practiced widely. Further study of portion size effects that exercise a wider range of food products, more diverse populations, and longer time periods could make an important contribution to better understanding potential environment risks for excess energy intake.

Aspects of this study that particularly intrigued us were 1) size and stability of the portion size effect on energy intake with very limited control of extraneous factors such as where, when and with whom people ate their lunches; 2) the very weak compensation for single meal overfeeding in our participants over a fairly long period of time; and 3) the relative ease and low cost of this experimental procedure. Overall, we believe that these observations indicate that studies of food exposures in the natural environment are very feasible and could provide important data for informing possible food policies designed to address obesity.

## Conclusion

Food portion size is a readily modified characteristic of the environment. Therefore, the potential for large food portion sizes to promote sustained increases in energy intake and conversely small food portion sizes to reduce energy intake deserves more study.

## Competing interests

The author(s) declare that they have no competing interests.

## Authors' contributions

RWJ contributed to the design of the study, was the primary writer of the manuscript, and was involved in the analysis and interpretation of the data. SR coordinated the implementation of the project, recruited participants, collected and analyzed data, and wrote up the methods section of the manuscript. CLD contributed to the conceptualization and design of the experiment and the development of measures and diet intervention components. LJH provided assistance with study design, data collection, and data analysis. ASL contributed to the design and rationale of study. PRP provided consultation and assistance with recruitment and data collection. JEB contributed to data analysis and interpretation. All authors contributed to the manuscript writing.
